# Cause-Effect: The Relationship between Role and Experience with Psychological and Physical Responses in the Competition Context in Soccer Referees

**DOI:** 10.5114/jhk/169174

**Published:** 2023-07-25

**Authors:** Alfonso Castillo-Rodríguez, Jesús Lorenzo Rodríguez Caparrós, Antonio Figueiredo, Francisco Tomás González-Fernández, Wanesa Onetti-Onetti

**Affiliations:** 1 Department of Physical Education and Sports, Faculty of Sport Sciences, University of Granada, Granada, Spain.; 2 Research Unit for Sport and Physical Activity, Faculty of Sport Sciences and Physical Education, University of Coimbra, Coimbra, Portugal.; 3 Faculty of Education, Universidad Internacional de La Rioja, Logroño, Spain.

**Keywords:** self-esteem, self-confidence, impulsivity, anxiety, performance, GPS

## Abstract

This study analyzed the effects of psychological responses (impulsivity, self-esteem, self-confidence, and anxiety) in baseline and pre-competitive contexts of national soccer referees (SRs), their control of the game (yellow and red cards), and physical performance (PP) according to the role (assistant or main referee), and experience in the category. Twenty-seven national SRs from Spain participated in this study. Baseline and pre-competitive psychological data were collected through the Competitive State Anxiety Inventory-2 (CSAI-2 test), Urgency, Premeditation (lack of), Perseverance (lack of), Sensation Seeking, Positive Urgency, Impulsive Behavior Scale (UPPS-P test), and Rosenberg tests. Furthermore, WIMU PRO™ inertial devices were used to monitor PP. The results indicated that a lack of perseverance and self-esteem were higher in the main SRs and somatic anxiety in assistant SRs. Experience of SRs was related to anxiety, self-confidence, self-esteem, impulsivity dimensions, and PP metrics (p < 0.05). Finally, red cards were related to positive and negative urgency (r = 0.38 and r = 0.35, p < 0.05, respectively). In conclusion, the main SRs and SRs with more experience had better psychological characteristics and PP in a competitive context. However, yellow and red cards were not associated with these factors, although red cards were related with urgency. Based on these data, specific training programs could be incorporated to enhance emotional control in SRs with less experience to achieve greater performance and professional development.

## Introduction

Soccer is a complex sport, involving numerous factors (e.g., physical, biomechanical, physiological, nutritional, psychological, etc.; Mackenzie and Cushion, 2013). Physical performance (PP) has been extensively investigated in all soccer player populations depending on expertise levels, experience, categories, and age (Walker et al., 2019). Recently, PP in competition has increased substantially, resulting in a better fitness level of soccer players competing at a high level (Bradley et al., 2016). This has also occurred in soccer referees (SRs) as an intrinsic part of the game. Studies have explored elements that substantially influence SR preparation, such as physical condition and body composition (Castillo-Rodríguez et al., 2021a), nutritional care (Jenner et al., 2019; Montesano et al., 2019), regulated and personalized training, and the control of psychological variables (Castillo-Rodríguez et al., 2021b; Montesano et al., 2019; Muñoz-Arjona and Castillo-Rodríguez, 2020; Muñoz-Arjona et al., 2022; Włodarczyk et al., 2011). These factors have different influences on SR decision making during competition, one of the most relevant being emotional control (Muñoz-Arjona and Castillo-Rodríguez, 2020; Weinberg and Richardson, 1990).

### Body and Mind: The Referee’s Preparation

The study of psychological variables (e.g., motivation, stress, anxiety, self-confidence [SC], mood states, self-esteem [SE], fear of social evaluation, etc.; Coudevylle et al., 2011; Hammermeister and Burton, 2001), and cognitive variables (e.g., attention, executive functions, vigilance, impulsivity, etc.; Castillo-Rodríguez et al., 2018; López-Aguilar et al., 2022; López-Aguilar et al., 2021a), along with their incidence in PP, have clarified how their strength or weight could guarantee athletes’ success (Muñoz-Arjona and Castillo-Rodríguez, 2020). Impulsivity is a construct that presents a multidimensional character, associated with sensation seeking and novelty (Zuckerman et al., 1993), pursuing small and immediate rewards (Cherek and Lane, 1999; López-Aguilar et al., 2021b). Anxiety is an important construct (Junge and Feddermann-Demont, 2016) and is divided into cognitive anxiety (CA), corresponding to the difficulty in maintaining concentration, and somatic anxiety (SA), referring to the perception of bodily symptoms caused by autonomic nervous system activation, such as an accelerated heart rate and sweating (Grossbard et al., 2009; Martens et al., 1990). Both types of anxiety influence PP differently (Muñoz-Arjona and Castillo-Rodríguez, 2020), confirming the existence of a controversy with the type of the relationship. No relationship between CA and PP was found in similar athletes (Rodrigo et al., 1990; Kais and Raudsepp, 2005), while others observed a negative linear and an inverted-U relationship between SA and PP (optimal PP for medium SA values and lower PP for low and high SA values) (Craft et al., 2003; Martens et al., 1990; Woodman and Hardy, 2003). Other authors found a direct relationship between CA and PP (Jones and Swain, 1992; Jones et al., 1993; Mellalieu et al., 2004) and an inverse relationship between SA and PP (Muñoz-Arjona and Castillo-Rodríguez, 2020).

SE is related to physical and psychological health (Rosenberg, 1965) and positively associated with athletes’ mental well-being due to its relationship with positive psychological (e.g., resilience) and emotional (e.g., fear, anxiety, depression, etc.) characteristics, and evaluation in stressful situations (Baumeister, 1993; González-Hernández et al., 2023). Athletes with low SE levels perceived sports competition as threatening, while those with high SE levels perceived it as demanding, obtaining a strong relationship between SE and PP (Muñoz-Arjona and Castillo-Rodríguez, 2020). Regarding SC, defined as the degree of certainty that athletes possess in relation to their ability to succeed in sports (Vealey, 1986), numerous investigations have revealed its impact on sports practice (Bačanac, 2014), traditionally finding positive relationships between SC and PP (Chamberlain and Hale, 2007).

### Decision Making and Control of the Competition

Multiple factors, such as experience in sports competition, can modulate athletes’ emotional states. Such experience is related to mastering various psychological skills (Rosnet, 2000), with the most experienced athletes having greater emotional control (Hanton et al., 2008). In semi-professional SRs, there is a negative relationship between experience and anxiety (Muñoz-Arjona and Castillo-Rodríguez, 2020), although this study focuses on category promotion tests. However, novice SRs offer higher rates of motivation and SC, affirming that competitive age or experience influence the athlete and consequently the SR, indicating that SC increases with competitive experience (Bačanac, 2014).

Considering PP, it has been observed that in competition, a soccer player runs an average of ~11 km in different speed ranges, mostly at low intensities (walking, jogging, etc.) or standing, wherein the aerobic system predominates (Bradley et al., 2013); this is consistent with values in amateur SRs (Castillo-Rodríguez et al., 2021a). However, soccer’s evolution has led to a significant increase in these demands, focusing on greater explosive actions of maximal/submaximal intensities (accelerations and decelerations), performed at high intensity that can accumulate muscle fatigue, which could affect decision making (Castillo et al., 2018). This quantification is relevant for designing a training program that is crucial for optimizing PP and preventing the occurrence of injuries (Bradley et al., 2016). Currently, the GPS is the most reliable monitoring device (Malone et al., 2017; Owen et al., 2017), offering information on multiple variables, such as total distance covered, high-intensity activities, speed thresholds, and other physiological and metabolic metrics.

### The Present Study

Therefore, the initial hypotheses of this study were that the experience of SRs would show a positive correlation with positive psychological skills such as SE and SC (H1a), and a negative correlation with negative psychological variables such as impulsivity and anxiety (H1b), and with the number of yellow/red cards shown in the competition (H1c). In addition, it was considered that the role would also affect the variance of psychological responses, e.g., anxiety and self-esteem, among others, since the main SRs have greater responsibility in the decision making of the competition and develop a greater PP than assistant SRs (H2). Therefore, the aim of this study was to analyze psychological responses (impulsivity, SE, CA, SA, and SC) prior to the competition, and PP and yellow/red cards shown by semi-professional SRs, considering their role and experience.

## Methods

### Participants

Twenty-seven national SRs participated voluntarily in the present study. Age, body mass, body height, and experience were 28.9 ± 4.92 years; 70.2 ± 8 kg; 175.7 ± 5.52 cm and 4.9 ± 3.3 years, respectively. Data were collected on physical and psychological responses in ten matches during the 2021–2022 season. All the main and assistant SRs passed the corresponding physical tests within their categories (September, December, and April). Participants were informed of the procedures, objectives, methodology, benefits, and potential risks of the study. The inclusion criteria were that participating SRs had already passed their physical tests that enabled them to referee, and additionally SRs must not have had acute or chronic illnesses in the previous two months that could influence their performance.

### Informed Consent

This study followed the principles of the Declaration of Helsinki (2013) and was approved by the Ethics Committee of the University of Granada (471/CEIH/2018). Participants were informed of the study aims, methods, and procedures. They provided written informed consent for inclusion of personal and fully anonymized material.

### Measures

First, the short version of the UPPS-P impulsivity questionnaire (Spanish version of Cándido et al., 2012), developed by Whiteside et al. (2005) and containing 20 items, was completed. The questionnaire differentiated between five dimensions of impulsivity: positive urgency (Urg+; e.g., “I act rashly when experiencing positive emotions”), negative urgency (Urg-; e.g., “I act rashly when experiencing negative emotions”), lack of premeditation PRE (e.g., “lack of consideration of the consequences my decisions will have”), lack of perseverance (PER; e.g., “tendency to stop doing tasks that I find boring or demanding”), and sensation seeking (SS; e.g., “willingness to engage in novel activities”). The psychometric properties of the Spanish version are adequate, with Cohen’s reliability scores ranging from 0.61 to 0.81 (Cándido et al., 2012).

Second, the Rosenberg test was completed to determine the SE degree (Atienza et al., 2000; Rosenberg, 1965). It comprises 10 items (five positive and five negative), with a 4-point Likert scale, ranging from 1 = strongly agree to 4 = strongly disagree. When obtaining the total score, <25 points indicated low SE; 26–29 points indicated average SE; and 30–40 points indicated high (adequate) SE.

Third, the Competitive State Anxiety Inventory-2 (CSAI-2) questionnaire by Martens et al. (1990) determined AC, SA, and SC levels. It comprises 27 items assessed on a 4-point Likert scale: 1 = nothing, 2 = something, 3 = quiet, 4 = a lot. To evaluate each subscale, measurements of corresponding items were considered.

Finally, WIMU PRO™ inertial devices (RealTrack Systems, Almería, Spain) were used to monitor PP. These devices present high precision and reliability for variables such as speed or distance, with a 1 GHz microprocessor (Bastida-Castillo et al., 2018). Furthermore, the measurement of multiple variables, using accelerometers and location, is frequently used to quantify PP in soccer (Pons et al., 2021). For this study, distance variables were analyzed, establishing five categories/speed zones: walking distance (WalkD), 0–5.9 km•h^−1^; low-speed running distance (LSRD), 6–12 km•h^−1^; medium-speed running distance (MSRD), 12–18 km•h^−1^; high-speed running distance (HSRD), 18–21 km•h^−1^; very high-speed running distance (VHSRD), 21–24 km•h^−1^; and sprint distance (SPD), higher than 24 km•h^−1^.

### Design and Procedures

**Figure 1:**
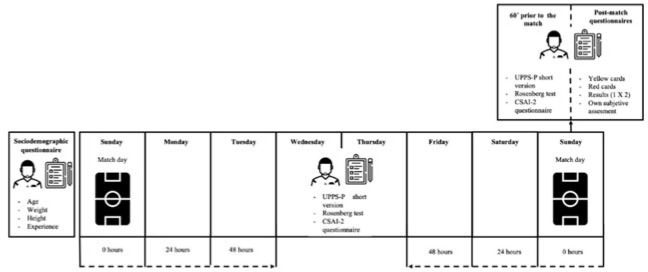
Schematic representation of the study.

This study had a cross-sectional, quasi-experimental design, and was carried out from September 2021 to May 2022. First, the Technical Committee of Referees was contacted, and subsequently, the interested participants were contacted through email. After selecting the participants and explaining the objectives, methodology, and protocols of the research, a sociodemographic questionnaire ad-hoc was administered to collect information on body mass, height, age, experience in the national category, and experience at previous levels. Then, the CSAI-2, UPPS-P, and the Rosenberg tests were administered 48 hours after the last match and 48 hours before the next one to determine the referees’ basal psychological states. Subsequently, on the match day, the same tests were administered 60 min prior to the match. WIMU PRO™ inertial devices were worn during the match to record PP. Finally, SRs completed a post-match questionnaire recording the number of yellow and red cards shown, the result (1 X 2), and their own subjective assessment of the difficulty of refereeing from 0 to 10 (Figure 1).

The dependent variables in this study were sociodemographic characteristics (questionnaire), psychological, baseline, and pre-competitive characteristics (CSAI-2, UPPS-P, and Rosenberg tests), post-competition characteristics (questionnaire), and PP through the WIMU PRO™ inertial devices. The independent variables were experience and the role of the SR.

### Statistical Analysis

Statistical programs SPSS for Windows version 23 (IBM SPSS Statistics, Chicago, USA) and Microsoft Office Excel (Microsoft Corp., Redmond, Washington, DC, IL, USA) were used. The Shapiro-Wilk test found that variables followed a normal distribution. Tests were analyzed for independent samples (*t*-test), with the referee role (main or assistant) as an independent variable. The effect size for the *t*-test was interpreted using Cohen’s *d* values. The following criteria were used to interpret the effect size: small effect (*d* < 0.20), moderate effect (0.20 ≤ *d* < 0.80), and large effect (*d* ≥ 0.80) (O’ Donoghue, 2013). Finally, Pearson’s correlation and simple linear regression tests were performed between experience and psychological responses, PP, and the number of cards shown in the competition. The level of significance was set at *p* < 0.05.

## Results

A mean comparison test with an independent variable (*t*-test) was performed to determine differences in PP (with relative values) depending on the role played in the competition (Table 1). There was an obvious difference between the main and assistant SRs. For these reasons, the evaluation of psychological skills was carried out considering the role played.

**Table 1:** Physical performance according to the SR role.

Physical performance	Main SR (n = 9)	Assistant SR (n = 18)	*t*	*p*	*d*
Sprints (events•min^−1^)	4.22 **±** 3.53	0.67 **±** 1.09	3.983	< 0.001	1.626
Accelerations (events•min^−1^)	1.61 **±** 0.32	1.00 **±** 0.29	4.956	< 0.001	2.023
Decelerations (events•min^−1^)	1.44 **±** 0.36	1.04 **±** 0.35	2.766	0.011	1.129
Maximum speed (km•h^−1^)	26.23 **±** 2.57	24.09 **±** 1.74	2.573	0.016	1.051
Total distance (m•min^−1^)	100.3 **±** 7.36	56.39 **±** 6.58	15.739	< 0.001	6.426
WalkD (m•min^−1^)	38.92 **±** 1.17	31.77 **±** 3.50	5.915	< 0.001	2.415
LSRD (m•min^−1^)	41.87 **±** 4.46	18.80 **±** 4.85	11.955	< 0.001	4.880
MSRD (m•min^−1^)	12.95 **±** 2.48	4.19 **±** 1.51	11.425	< 0.001	4.664
HSRD (m•min^−1^)	4.30 **±** 1.55	1.14 **±** 0.69	7.410	< 0.001	3.025
VHSRD (m•min^−1^)	1.76 **±** 1.37	0.40 **±** 0.31	4.082	< 0.001	1.666
SPD (m•min^−1^)	0.55 **±** 0.53	0.08 **±** 0.17	3.462	0.002	1.413

WalkD: Walking distance covered from 0 to 6 km•h^−1^; LSRD: low-speed running distance covered from 6.1 to 12 km•h^−1^; MSRD: running distance at medium speed covered from 12 to 18 km•h^−1^; HSRD: high-speed running distance covered from 18 to 21 km•h^−1^; VHSRD: distance travelled at very high speed covered from 21 to 24 km•h^−1^; SPD: sprint race distance covered at speed higher than 24 km•h^−1^

Subsequently, both baseline and pre-competitive psychological responses were analyzed according to the role (Table 2). Differences were found both at baseline in SE and lack of perseverance (*p* < 0.05; *d* > 1.2), as in the pre-competitive moment in SE and SA (*p* < 0.05; *d* > 0.8). The attending SRs showed higher SA at the pre-competitive time, less perseverance at baseline, and lower SE at both times.

**Table 2:** Psychological responses according to the SR role.

Context	Psychological responses	Main SR (n = 9)	Assistant SR (n = 18)	*α*	*t*	*p*	*d*
Basal	SE	31.5 ± 3.00	28.1 ± 2.67	0.949	1.923	0.054	1.205
	URG-	9.75 ± 3.20	12.3 ± 3.20	0.871	−1.264	0.238	−0.792
	URG+	9.75 ± 1.26	10.7 ± 3.86	0.665	−0.476	0.646	−0.298
	PRE	10.0 ± 2.83	7.71 ± 3.59	0.907	1.086	0.306	0.681
	PER	11.3 ± 2.63	5.57 ± 1.40	0.961	4.770	0.001	2.990
	SS	9.50 ± 3.11	8.86 ± 3.13	0.758	0.328	0.750	0.206
	Σ impulsivity	50.3 ± 4.90	45.1 ± 9.86	0.812	0.953	0.365	0.597
	CA	9.50 ± 3.32	7.14 ± 1.95	0.888	1.510	0.165	0.946
	SA	10.5 ± 5.00	8.86 ± 2.41	0.809	0.750	0.472	0.470
	SC	17.5 ± 3.70	19.0 ± 1.73	0.775	−0.935	0.374	−0.586
Pre-competition	SE	32.9 ± 3.76	29.2 ± 2.50	0.944	3.078	0.005	1.257
	URG-	11.8 ± 4.24	12.4 ± 2.52	0.846	−0.472	0.641	−0.193
	URG+	10.9 ± 2.71	11.4 ± 2.33	0.796	−0.498	0.623	−0.203
	PRE	8.11 ± 4.17	7.56 ± 3.17	0.909	0.387	0.702	0.158
	PER	8.00 ± 4.64	6.39 ± 3.01	0.885	1.092	0.285	0.446
	SS	10.1 ± 3.48	10.2 ± 2.82	0.813	−0.089	0.930	−0.036
	Σ impulsivity	48.9 ± 5.35	47.9 ± 4,02	0.850	0.515	0.611	0.210
	CA	6.89 ± 2.47	8.50 ± 3.92	0.896	−1.122	0.273	−0.458
	SA	8.11 ± 2.62	10.8 ± 3.14	0.792	−2.192	0.038	−0.895
	SC	19.2 ± 1.72	19.1 ± 1.26	0.811	0.287	0.776	0.117

SE: Self-esteem; URG+: positive urgency; URG−: negative urgency; PRE: lack of premeditation; PER: lack of perseverance; SS: sensation seeking; Σ impulsivity: unidimensional impulsivity; CA: cognitive anxiety; SA: somatic anxiety; SC: self-confidence

*T*-tests were performed for related samples, and no significant differences were found between baseline and pre-competitive moments. Finally, correlations between the impulsivity variables and data on perceived difficulty and the cards shown were analyzed (Table 3). Perceived difficulty was inversely correlated with lack of perseverance (*r* = −0.456, *p* < 0.01). In addition, positive and negative urgency were directly correlated with the number of red cards shown in the match.

**Table 3:** Correlations between impulsivity, displayed cards and perceived difficulty.

	URG-	URG+	PRE	PER	SS	Σ impulsivity
**Yellow cards**	0.056	0.239	−0.094	−0.003	−0.049	0.322
**Red cards**	0.352^*^	0.379^*^	−0.109	−0.327	−0.037	0.183
**Difficulty of SR**	0.205	0.259	−0.030	−0.456^**^	−0.090	0.019

* p < 0.05; ^**^ p < 0.01; URG+: positive urgency; URG−: negative urgency; PRE: lack of premeditation; PER: lack of perseverance; SS: sensation seeking; Σ impulsivity: unidimensional impulsivity

The correlation of experience with psychological, physical and specific responses to the match were evaluated (Table 4). With respect to the baseline, experience correlated directly with medium-high levels of SE, PER, and SS (*r* = 0.64 to 0.92; *p* < 0.05). Regarding the pre-competitive moment, there were moderate and high direct correlations with SC and SE, respectively, and inverse moderate correlations with CA and SA. During the competition, it was proven that the experience of SRs correlated directly with the total distance covered, decelerations, and distances covered from 6 to 21 km•h^−1^ (*p* < 0.05).

**Table 4:** Single linear regression tests between SR experience and psychological responses, physical performance, cards and match difficulty.

Moment	Variable	Beta	(95% CI)	*β*	*p*
Baseline (absolute data)	SE	0.901	(0.607; 1.195)	0.918^**^	0.000
	URG−			−0.341	0.152
	URG+			−0.150	0.330
	PRE			0.228	0.251
	PER	0.792	(0.262; 1.322)	0.748^**^	0.004
	SS	0.600	(0.061; 1.138)	0.643^*^	0.033
	Σ impulsivity			0.426	0.095
	CA			0.225	0.253
	SC	−0.368	(−0.900; 0.164)	−0.463	0.053
	SA	0.610	(−0.051; 1.272)	0.571^*^	0.033
Pre-competitive (absolute data)	SE	0.898	(0.688; 1.109)	0.870^**^	0.000
	URG−			−0.251	0.104
	URG+			−0.111	0.291
	PRE			0.013	0.474
	PER			0.266	0.090
	SS	0.310	(−.041; .660)	0.342^*^	0.040
	Σ impulsivity			−0.221	0.134
	CA	−0.484	(−.877; −.090)	−0.452^**^	0.009
	SC	0.171	(.012; .330)	0.404^*^	0.018
	SA	−0.471	(−818; −.123)	−0.487^**^	0.005
During the competition (relative data)	Total distance	3.340	(0.942; 5.737)	0.454^**^	0.004
	WalkD	0.492	(−0.031; 1.015)	0.361^*^	0.032
	LSRD	1.700	(0.375; 3.024)	0.467^**^	0.007
	MSRD	0.712	(0.220; 10.203)	0.512^**^	0.003
	HSRD	0.286	(0.090; 0.481)	0.516^**^	0.003
	VHSRD	0.115	(−0.005; 0.235)	0.367^*^	0.030
	SPD	0.036	(−0.011; 0.083)	0.307	0.065
	Accelerations	0.046	(−0.002; 0.095)	0.368^*^	0.030
	Decelerations	0.049	(0.004; 0.094)	0.411^*^	0.017
	Sprints	0.003	(0.000; 0.007)	0.376^*^	0.029
	Maximum speed			0.283	0.076
Post-competitive (absolute data)	Yellow Cards			0.191	0.170
	Red Cards			0.230	0.124
	Match difficulty			0.121	0.273

Note: ^*^ p < 0.05; ^**^ p < 0.01; Beta: non standardized coefficient; β: standardized regression coefficient; WalkD: walking distance covered at speed from 0 to 6 km•h^−1^; LSRD: low-speed running distance covered from 6.1 to 12 km•h^−1^; MSRD: running distance covered at medium speed from 12 to 18 km•h^−1^; HSRD: high-speed running distance covered from 18 to 21 km•h^−1^; VHSRD: distance covered at very high speed from 21 to 24 km•h^−1^; SPD: sprint race distance covered at speed higher than 24 km•h^−1^;

SE: self-esteem; URG+: positive urgency; URG−: negative urgency; PRE: lack of premeditation; PER: lack of perseverance; SS: sensation seeking; Σ impulsivity: unidimensional impulsivity; CA: cognitive anxiety; SA: somatic anxiety; SC: self-confidence

## Discussion

This study analyzed psychological responses (impulsivity, SE, CA, SA, and SC) prior to and after competition, as well as the PP of semi-professional SRs, considering their role and experience. The hypotheses raised confirmed that positive psychological skills such as SE and SC had a direct relationship with the experience of SRs (H1a) and an inverse relationship with negative psychological variables such as impulsivity and anxiety (H1b) as well as with the number of cards shown in the competition (H1c).

The results showed that not all SRs of the same category had similar psychological responses previous to competition, but that they varied based on their experience in refereeing. According to studies by Hanton et al. (2008), Muñoz-Arjona and Castillo-Rodríguez (2020), and Rosnet (2000), SRs with greater experience present lower CA and SA, while higher SC and SE. These findings mean that SRs are in a better condition for competition (Muñoz-Arjona and Castillo-Rodríguez, 2020). This entails an improvement in PP of SRs. Authors such as Weinberg and Richardson (1990) argue that more experienced athletes offer greater emotional control, making calmer and safer decisions.

In turn, no differences were found in the responses of SC, SE, CA, and SA in data collection between the baseline and pre-competition conditions, presenting high values of SC and SE, and lower values in CA and SA. This stability in the baseline and pre-competitive data collection indicates that SRs participating in this study would face the competition with a positive state of confidence in tackling different competitive situations. Another study conducted by López-Aguilar et al. (2021a) analyzed the relationship between the pre-competition psychological variables of semi-professional SRs and their physical-physiological responses during competition, and compared these responses according to their level of experience. The results showed that experienced SRs presented lower scores in negative psychological responses and higher scores in positive ones, revealing correlations between SE, CA, and SC with physical-physiological responses. Experienced SRs had fewer negative psychological responses (CA and SA) and higher positive responses (SC and SE) than non-experienced referees.

Other studies have compared PP depending on the main or assistant SR role. An example is a study by Barbero et al. (2012) on PP of field SRs in the Copa America. Those authors concluded that PP of the main SRs was higher than that of assistants. In addition, they equated these demands with those of European professional SRs. In the current study, comparing the main and assistant SR roles, it was proven that the role of the main SR determined a different PP to that of the assistant SR. Although this may be evident, by relativizing the data and focusing on objective aspects, it can be said that the main SRs perform more physically intense work than the attending SRs. When maximum speed reached by the SR during the match is analyzed, higher maximum speed is observed in the main SRs than in assistants, as is the case with other values such as the sprint distance (>24 km•h^−1^). This suggests that the physical condition and PP of the main SRs are higher during a soccer match than those demanded of the assistant SRs.

Similarly, we analyzed both basal and pre-competitive psychological responses depending on the role played. We found significant differences both at baseline in SE variables and lack of perseverance (*p* < 0.05; *d* > 1.2), as well as in the pre-competitive moment in SE and SA variables (*p* < 0.05; *d* > 0.8). Considering self-efficacy, the attending SRs showed higher SA at the pre-competitive time, less perseverance at baseline, and lower SE at both times (López et al., 2022). In addition, the impulsivity dimensions of positive and negative urgency were correlated with the number of red cards shown. This finding is novel in science, as the relationship of precompetitive impulsivity dimensions with the number of cards shown during competition has not been investigated to date. However, it is known that most of the non-relevant decisions or simple decisions, of which the impact on the competition is low, the level of precision is very high in SRs (Samuel et al., 2019), thus it is necessary to determine in the future the success and error rate of difficult decisions or with high impact on the competition. In the present study, the correlation between experience and psychological and physical responses was evaluated. With respect to baseline evaluations, experience correlated directly with medium-high levels of SE, PER, and SS (*r* = 0.64 to 0.92; *p* < 0.05). Regarding the pre-competitive moment, as we have seen above, there were moderate and high direct correlations with SC and SE, respectively, and inverse, moderate correlations with CA and SA. During the competition, it was proven that the experience of SRs correlated directly with the total distance covered, decelerations, and distances covered at speed from 6 to 21 km•h^−1^ (*p* < 0.05). This leads to several conclusions. It is true that the experience of SRs has a positive influence on their SC and SE and a negative influence on CA and SA. This may be because as SRs develop their work year after year, they are trained in the emotional management of the difficulties that may be encountered during refereeing. When a SR manages to reach the category of 3^rd^ RFEF, they have passed a period of training, category by category, that allows not only a better emotional management of the matches, but also an acquisition of physical skills that make it possible for them to have high PP and a better placement and approach to the playing area, thus benefitting the decision-making process (Castillo-Rodríguez et al., 2021b).

### Strengths, Limitations, and Future Research Directions

This study has several limitations which should be acknowledged. First, the sample size was too small to present psychological variables and allow generalization. Therefore, the conclusions should be interpreted with caution. Semi-professional SRs that are constantly evaluated by informants hired to be able to promote them to a higher category do not accept to participate in this type of study, thus they present a limitation. In addition, it would be beneficial to recommend training programs in emotional management, such as mindfulness (Coimbra et al., 2021) to increase levels of SE and SC, control CA and SA levels, along with specific physical preparation that promotes their PP, to achieve maximum sporting excellence within the role of the SR. Future research should provide information on the variability in responses or fluctuations between the SRs themselves. This study has also several strengths. Psychological responses were described according to the role of participation, considering that PPs are very different.

## Conclusions

The main finding of this study is that SRs with more experience had higher levels of self-esteem and confidence, lower levels of cognitive and somatic anxiety, and a greater physical performance. The initial hypotheses confirmed that positive psychological skills were directly related to the experience of SRs and inversely related to negative psychological skills. The number of disciplinary sanctions given in the competition (yellow and red cards) was not associated with psychological skills, PP or experience factors.

## Implications for Practice

The Referees Committee could consider the referee's experience for assignment in competitive matches in which decision making could have a negative impact on teams, such as matches in which promotion/relegation and Champions League places are determined, among other consequences. In addition, to increase the experience and improve the pre-competitive psychological responses and characteristics of novice referees, further participation as an assistant referee in high-level competitions could be recommended to increase the referee's experience and ability to perceive and learn from the head referee. It may be useful for intervention programs for inexperienced novice referees to improve psychological skills critical to decision making and physical performance, such as impulsivity, anxiety, self-confidence, and self-esteem. Sports psychologists could help national SRs train mental skills, studied in individual sessions, through recruitment by the National Committee of Soccer Referees. For example, mindfulness has been shown to improve the ability to concentrate, thus future researchers or practitioners are urged to develop validated and reliable programs.
